# Holocene deglaciation drove rapid genetic diversification of Atlantic walrus

**DOI:** 10.1098/rspb.2023.1349

**Published:** 2023-09-27

**Authors:** Emily J. Ruiz-Puerta, Xénia Keighley, Sean P. A. Desjardins, Anne Birgitte Gotfredsen, Shyong En Pan, Bastiaan Star, Sanne Boessenkool, James H. Barrett, Morgan L. McCarthy, Liselotte W. Andersen, Erik W. Born, Lesley R. Howse, Paul Szpak, Snæbjörn Pálsson, Hilmar J. Malmquist, Scott Rufolo, Peter D. Jordan, Morten Tange Olsen

**Affiliations:** ^1^ Section for Molecular Ecology and Evolution, Globe Institute, Faculty of Health and Medical Sciences, University of Copenhagen, Øster Farimagsgade 5-7, 1353 Copenhagen Kobenhavn, Denmark; ^2^ Arctic Centre & Groningen Institute of Archaeology, Faculty of Arts, University of Groningen, PO Box 716, 9700 AS Groningen, The Netherlands; ^3^ The Bureau of Meteorology, The Treasury Building, Parkes Place West, Parkes, Australian Capital Territory 2600, Australia; ^4^ Palaeobiology Section, Canadian Museum of Nature, PO Box 3443, Station D, Ottawa, Ontario, Canada K1P 6P4; ^5^ Section for GeoGenetics, Globe Institute, University of Copenhagen, Øster Voldgade 5-7, 1350 Copenhagen Kobenhavn, Denmark; ^6^ Centre for Ecological and Evolutionary Synthesis, Department of Biosciences, University of Oslo, Blindernveien 31, 0371 Oslo, Norway; ^7^ Department of Archaeology and Cultural History, NTNU University Museum, 7491 Trondheim, Norway; ^8^ McDonald Institute for Archaeological Research, Department of Archaeology, University of Cambridge, Downing Street, Cambridge CB2 3ER, UK; ^9^ Department of Ecoscience, Aarhus University, CF Møllers Allé 4-8, build. 1110, 8000 Aarhus C, Denmark; ^10^ Greenland Institute of Natural Resources, PO Box 570, 3900 Nuuk, Greenland; ^11^ Archaeology Centre, University of Toronto, 19 Ursula Franklin Street, Toronto, Ontario Canada M5S 2S2; ^12^ Department of Anthropology, Trent University, 1600 West Bank Drive, Peterborough, Ontario, Canada K9L 0G2; ^13^ Faculty of Life and Environmental Sciences, University of Iceland, Askja, Sturlugata 7, 101 Reykjavik, Iceland; ^14^ Icelandic Museum of Natural History, Suðurlandsbraut 24, 108 Reykjavík, Iceland; ^15^ Department of Archaeology and Ancient History, Lund University, Helgonavägen 3, 223 62 Lund, Sweden; ^16^ Global Station for Indigenous Studies and Cultural Diversity (GSI), GI-CoRE, Hokkaido University, Japan; ^17^ Natural History Museum of Denmark, University of Copenhagen, Denmark

**Keywords:** palaeogenetics, *Odobenus rosmarus rosmarus*, ancient DNA, Arctic, environmental change, marine mammals

## Abstract

Rapid global warming is severely impacting Arctic ecosystems and is predicted to transform the abundance, distribution and genetic diversity of Arctic species, though these linkages are poorly understood. We address this gap in knowledge using palaeogenomics to examine how earlier periods of global warming influenced the genetic diversity of Atlantic walrus (*Odobenus rosmarus rosmarus*), a species closely associated with sea ice and shallow-water habitats. We analysed 82 ancient and historical Atlantic walrus mitochondrial genomes (mitogenomes), including now-extinct populations in Iceland and the Canadian Maritimes, to reconstruct the Atlantic walrus' response to Arctic deglaciation. Our results demonstrate that the phylogeography and genetic diversity of Atlantic walrus populations was initially shaped by the last glacial maximum (LGM), surviving in distinct glacial refugia, and subsequently expanding rapidly in multiple migration waves during the late Pleistocene and early Holocene. The timing of diversification and establishment of distinct populations corresponds closely with the chronology of the glacial retreat, pointing to a strong link between walrus phylogeography and sea ice. Our results indicate that accelerated ice loss in the modern Arctic may trigger further dispersal events, likely increasing the connectivity of northern stocks while isolating more southerly stocks putatively caught in small pockets of suitable habitat.

## 1. Introduction

The Arctic is currently warming at rates well above the global average [1]. It has been predicted that this will ultimately lead to changes in the Arctic marine ecosystem composition and trophic networks [2–5], including northward-range shifts of marine species [6], altered foraging and haul-out behaviour [7], the introduction of novel pathogens [8] and putative species hybridizations [9]. However, it is generally unclear to what extent and how fast warming might affect Arctic marine organisms, and in particular, their population connectivity, diversity and extinction risk. In an attempt to predict the future effects of ongoing climate change, researchers are endeavouring to better understand the effects of past environmental changes—particularly, the last glacial maximum (LGM; 26.5–19.0 thousand years (ky) BP) and the subsequent Holocene deglaciation (11.7–6.0 ky BP) [10].

Marine mammals are often viewed as indicators of environmental change and overall ecosystem health in the Arctic [11]. Genetic analyses of Arctic marine mammals, such as bowhead whales (*Balaena mysticetus*) [12], narwhals (*Monodon monoceros*) [13–15], belugas (*Delphinapterus leucas*) [16] and polar bears (*Ursus maritimus*) [17,18], have revealed relatively low levels of genetic diversity, with most intraspecific differentiation attributed to allopatric divergence during and after the LGM. Thus, the most prevalent hypothesis is that Arctic marine mammals follow a *tabula rasa* scenario, in which they survived the LGM in southerly refugia, and recolonized the Arctic at the onset of Holocene warming [19]. However, Arctic pinnipeds, such as harp seals (*Pagophilus groenlandicus*) [20] and ringed seals (*Pusa hispida*) [21] are characterized by high levels of genetic diversity, comprising multiple distinct mitochondrial clades that appear to predate the LGM and have no clear geographical pattern. This indicates that they survived glaciations in high-latitude Arctic refugia, such as local polynyas or glacial fronts; i.e. the marine equivalent of the terrestrial *nunatak* scenario. Moreover, signatures of pre-LGM divergence and glacial survival in high-latitude refugia have been reported across multiple other Arctic marine organisms, including fish, invertebrates and macroalgae [22–25]. Evidently, the manner and pace that past environmental change has shaped the genetic diversity, abundance and distribution of Arctic marine biota is highly complex, possibly involving both *tabula rasa* and *polynya* scenarios and multiple waves of recolonization upon deglaciation. This complicates efforts to understand and mitigate the effects of ongoing global warming and associated human activities in the Arctic.

The walrus is a large-bodied pinniped with a pan-Arctic distribution, feeding mainly on bottom-dwelling molluscs and occupying areas characterized by shallow waters and access to suitable haul-out sites on sea-ice or land [26]. These characteristics make the walrus a key species in the Arctic marine environment, and consequently, it is often used as an indicator by non- and inter-governmental organizations (e.g. World Wildlife Fund (WWF), Conservation of Arctic Flora and Fauna (CAFF), North Atlantic Marine Mammal Commission (NAMMCO)) of the effects of environmental change and human activities. The walrus is currently divided into two subspecies, of which the Pacific (*O. r. divergens*) appears to be largely panmictic [27–29], whereas the Atlantic subspecies (*O. r. rosmarus*) consists of multiple genetically distinct populations [30,31]. The relatively high degree of population structure in the Atlantic walrus is unique among Arctic pinnipeds and cetaceans, raising key questions as to the timing and mechanisms driving these patterns. Divergence in North Atlantic walruses has been estimated at 145 thousand years ago (kya) for Canadian populations [32] and 268 kya for Northeast Atlantic-Pechora Sea populations [31], whereas the first estimate across most of the North Atlantic range was provided by Star *et al*. [33] at 251–23 kya, and later by Keighley *et al*. [34] at approximately 21 kya. In terms of glacial refugia, Born *et al*. [35] hypothesized the existence of a common ancestral walrus population in the North Atlantic about 12 kya, while Star *et al*. [33] found support for the existence of two major Atlantic mitochondrial clades—a western and an eastern (mixed) clade—and proposed that these reflect the existence of two separate LGM refugia at either side of the Atlantic Ocean.

Ultimately, despite much attention, it remains unclear whether the Atlantic walrus survived glacial periods in one, two or more refugia, where these refugia may have been located, at what speed the species expanded to recolonize its current Arctic range and how this shaped its genetic diversity. We harnessed the potential of ancient DNA (aDNA) and climate-proxy data to shed new light on the response of the Atlantic walrus to past environmental change. Specifically, we explored the spatio-temporal pattern of Atlantic walrus diversification through the phylogeographic analysis of 82 novel and previously published ancient and historical walrus mitogenomes [33,34], spanning a period of nearly 8000 years, and covering most of the Atlantic walrus' current range, as well as that of now-extinct populations (electronic supplementary material, table S1). We also used published climate-proxy data to map ice sheet coverage during selected periods of the LGM and subsequent Holocene warming, linking it to the chronology of Atlantic walrus diversification inferred from mitogenome data.

## 2. Material and methods

### (a) Zooarchaeological samples and existing data

To unravel the demographic and evolutionary history of Atlantic walrus populations, we screened 187 ancient and historical walrus specimens, selecting 82 with good coverage of the mitochondrial genome for genetic analyses (electronic supplementary material, table S1). Of these, 28 mitogenomes were from Keighley *et al*. [34], 10 were from Star *et al*. [33], whereas 44 mitogenomes were generated specifically for the present study. For the novel mitogenomes, specimens with seemingly good macroscopic preservation (i.e. not too degraded or porous) and from well-dated contexts (e.g. with radiocarbon dates or clear cultural affinity) were preferentially sampled. Additionally, when possible, samples from the same skeletal elements were chosen (e.g. left mandible) to avoid sampling the same individual more than once. The dates follow standard radiocarbon date reporting and are presented as BP indicating calibrated years before present (1950).

### (b) Preparation of ancient and historical Atlantic walrus remains

DNA laboratory work was undertaken at the Globe Institute, University of Copenhagen, Denmark. All samples were prepared in accordance with strict aDNA laboratory guidelines [36,37]. Specifically, all pre-amplification work was conducted in a specialized aDNA building, with rigorous cleaning and contamination control standards, including negative controls through extraction, library preparation and amplification. Bones were drilled to obtain 100–220 mg of fine bone powder, or cut up into 300 mg of small chunks. We used a Dremel hand drill (Micro 8050 and 4000) or an Osada dental drill (OS-40) and drill pieces included a dental Rosenbor for powder (sizes 012–031) or diamond cutter for chunks. The bone surface was first cleaned mechanically by drilling and discarding a thin layer of bone or tooth for all of the samples. Drilling was completed at the lowest possible speed (2000–5000 r.p.m.), and pauses were taken every few minutes to ensure that bones did not overheat and cause additional DNA degradation.

### (c) Ancient DNA extraction, targeted DNA enrichment and sequencing

aDNA was extracted following the protocol by Dabney *et al*. [38]. To increase the yield of endogenous DNA, bone chunks (but not powder) were subject to an initial bleach wash as per Boessenkool *et al*. [39]. Extracts were quantified using a High Sensitivity TapeStation (Agilent Technologies) before library build, following the Blunt-End-Single-Tube (BEST) protocol by Carøe *et al*. [40]. As per Barnett *et al*. [41], qPCRs were completed to determine the optimum number of cycles for amplification. Index reactions were 1 ul of 10 × Pfu Turbo Reaction Buffer (Agilent Technologies), 1.25 U of PfuTurbo Cx Hotstart DNA Polymerase (Agilent Technologies), 0.02 mg bovine serum albumin (BSA), 8.75 pmol each of a unique combination of forward and reverse indices (IDT) and 3.125 pmol of each deoxynucleotide triphosphate (dNTP). Thermal cycling conditions were an initial denaturing phase of 2 min at 95°C, followed by the annealing phase (cycles of 30 s at 94°C, 1 min at 57°C and 1 min at 68°C) and a final extension phase for 10 min at 70°C. In the qPCR reaction (Stratagene Mx 3 000), 1 ul of SYBRgreen fluorescent dye replaced 1 ul of water. For indexing, compatible 6 base pair hexamer motif indices were used. In addition, to maximize the capture of mitochondrial DNA from samples with low endogenous concentration, we used target-capture baits designed for marine mammal mitogenomes by Arbor Biosciences (https://arborbiosci.com/). Capture enrichment was performed following the manufacturer's instructions and library preparation and sequencing as described above.

Amplified libraries were purified and size selected with solid-phase reversible immobilization (SPRI) beads, targeting 60–600 base pairs (0.5× and 1.6× ratios). Samples with successful amplification following quantification on a High Sensitivity TapeStation were pooled together for sequencing in groups of at least 12 samples. Shotgun sequencing was performed on a range of Illumina technologies (MiSeq, HiSeq 2500 and HiSeq 4000) at the Danish National High-throughput Sequencing Centre, with read lengths of 80–150 bp and using either single or paired end protocols. Throughout all laboratory work, samples were randomly given a unique sample number, with different groupings for extraction, library build, amplification and sequencing to ensure no clustering of samples from a particular locality or time period. Samples run on the Illumina HiSeq 4000 were dual-indexed due to the risk of index-hopping [42].

### (d) Read alignment and filtering

The resulting raw sequenced data were analysed together with previously published raw sequence reads of Atlantic walrus [33,34] available from the European Nucleotide Archive (ENA) for all mitogenome analyses (electronic supplementary material, table S1). Reads were trimmed, filtered and aligned using the PALEOMIX (v.1.2.13.4) BAM pipeline [43]. An existing Atlantic walrus mitochondrial genome (National Center for Biotechnology Information (NCBI) accession: NC_004029.2) [44] was used as a mapping reference. The PALEOMIX pipeline [43] began by indexing raw reads and reference sequences, merging overlapping reads and identifying mate-pairs (for paired-end data) using SAMtools (v.1.9) [45] and bwa (v.0.7.17) [46]. MapDamage (v.2.0.9) [47] was used to assess the postmortem damage, confirming the authenticity of our aDNA. Adapter sequences, ambiguous, short sequences (less than 25 base pairs) and low-quality bases (Q ≤ 30) were removed with Adapter Removal (v.2.3.1) [48]. Output files were indexed and duplicates removed with SAMtools (v.1.3.1) [45] and MarkDuplicates (Broad Institute). Mitogenome haplotypes were called independently with ANGSD (v.0.921) [49] using SAMtools and BAQ computation [50] against the reference Atlantic walrus mitochondrial genome. Bases were not called for sites where depth of coverage was less than 3, reads were removed if there were multiple best hits during mapping, and the d-loop region was removed due to poor mapping against the reference mitogenome. Finally, we discarded sequences with less than 95% breadth of coverage, resulting in a dataset of 82 ancient and historical mitogenomes.

### (e) Genetic diversity and differentiation

DnaSP (v.6) [51] was used to estimate nucleotide and haplotype diversity, as well as the net number of nucleotide differences *d*_A_ for geographically determined groups of samples. Levels of genetic differentiation were quantified by *F*_ST_ estimates computed in Arlequin (v.3.5.2.2) [52] with 1023 permutations and correction for multiple testing following the Bonferroni method. A median-joining haplotype network was created in PopArt (v.1.7) [53].

### (f) Phylogenetic reconstruction

We first constructed an IQtree phylogeny [54] and analysed it in TempEst (v.1.5.3) [55] finding a positive correlation between genetic divergence and sampling time (*R*^2^ = 13.4), which indicates that the temporal signal in our data is sufficient to perform phylogenetic molecular clock analyses. Next, Bayesian mitogenome phylogenies were constructed using BEAST 2 (v.2.5.1) using the Pacific walrus mitogenome (NCBI GenBank accession GCA_000321225.1) as an outgroup. The program PartitionFinder v.2.1.1 [56] was used to determine the appropriate evolutionary model, gamma rate heterogeneity and invariable sites for the mitogenome sets. We tested both a strict clock model following previous studies on Atlantic walruses [33,57], as well as a relaxed clock exponential model (electronic supplementary material, tables S2–S4). Tree and clock models were linked for all the regions. The relaxed clock exponential model showed highest likelihood values and was hence selected for subsequent analyses [58,59]. We used sample tip-dates (i.e. the age of each specimen; also called ‘tip-dating’ or ‘sampled ancestors') to calibrate the BEAST2 phylogeny and infer divergence times [60]. Priors for effective population size (ePopSize), growth rate (growthRate) and uncorrelated exponential relaxed clock mean (ucedMean) was set at infinity as per BEAST2 default settings. The posteriors for these variables were estimated by BEAST2 based on the tip-dates and level of genetic variation in the data. The Markov chain Monte Carlo (MCMC) consisted of 80 million generations, with a pre-burn-in of 10%, and sampling for every 1000 for trees, screen logs and log files. Output files were checked for convergence in Tracer (v.1.7.1), ensuring a minimum effective sample size (ESS) values of greater than or equal to 200 [61]. Output tree files were analysed in TreeAnnotator (v.2.6.0) with a burn-in of 10% and a maximum clade credibility tree as target tree type and the phylogeny was visualized in FigTree (v.1.4.3) [62]. The BEAST2 input .xml file, and output files are provided as supplementary files. To supplement the Bayesian analyses, we also constructed a maximum-likelihood phylogenetic tree using IQtree [54] and the partitions, settings and evolutionary model suggested by Partition finder v.2.1.1.

### (g) Demographic modelling

In order to model the demographic history of Atlantic walruses, two Bayesian skyline plots (BSP) were generated using BEAST2. The first BSP consisted of the eastern (mixed) clade (*N* = 39), and the second BSP consisted of sequences belonging to the western clade (*N* = 36). We did not perform BSP analyses for the previously undescribed northwestern clade (see results), given its small sample size (*N* = 7). The BSPs made use of the input alignments and partitions from the phylogenetic analyses with an MCMC consisting of 50 million generations. Following the same methodology for the phylogenies, the output log and tree files were inspected and processed in Tracer (v.1.7.1).

### (h) Reconstruction of Holocene ice cover

In order to reconstruct LGM and Holocene ice cover and compare it with our demographic and evolutionary analyses, we created a series of maps with glacial ice cover for representative time periods in ArcGIS software by Esri [63] (v.10.8). We obtained information on ice extent from [64–66] and projected this into the contemporary distribution of Atlantic walrus stocks as defined by distributions taken from [57,67,68]. Figures were prepared in Inkscape (v.0.92) [69].

## 3. Results and discussion

### (a) Multiple Atlantic refugia during the last glacial maximum

**Figure 1.**
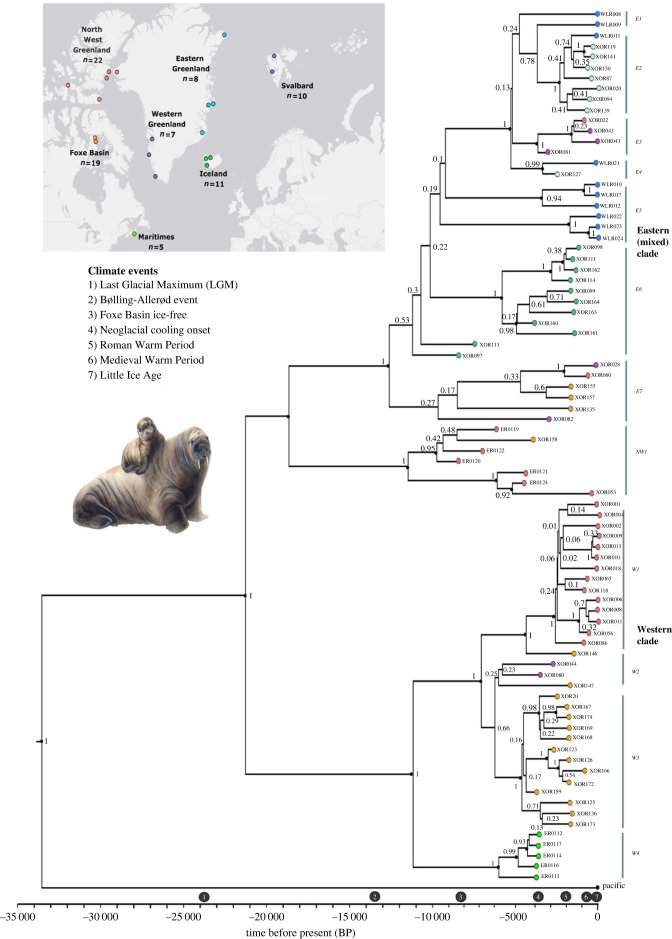
The divergence and radiation of the Atlantic walrus. Time-calibrated Bayesian phylogeny for 82 ancient and historical Atlantic walrus mitogenomes. Tips are colour-coded according to geographical origin corresponding to the map insert and placed in the phylogeny according to the age of the specimen. Posterior probability values are provided at each node with nodes greater than 80% of posterior support marked by black dots. The Pacific walrus was included as an outgroup and putative clade names (e.g. E1) are provided as reference for the discussion. Numbers 1–7 on the time scale refer to key climatic events in the North Atlantic. Walrus illustration by Elena Kakoshina (artkakos.com). All sample information is listed in electronic supplementary material, table S1, and additional details on the phylogenetic analyses provided in electronic supplementary material, figure S1 and tables S2–S4.

Our Bayesian time-calibrated phylogenetic analysis of 82 ancient and historic walrus mitogenomes shed new light on the Atlantic subspecies' phylogeography and past response to environmental change (figure 1; electronic supplementary material, figure S1 and table S2). The authenticity of our aDNA data, partition scheme, temporal signal and choice of clock and mutation models was supported by model testing as recommended by Rieux and Balloux [70] (Methods; electronic supplementary material, tables S3–S4), and the topology supported by maximum-likelihood phylogenetic analyses in IQtree (electronic supplementary material, figure S2). The phylogenetic analyses confirmed the existence of the two major evolutionary clades—a western and an eastern (mixed) clade—identified in previous studies of Atlantic walrus [33,34], and estimated their divergence to about 25.0 kya BP (95% highest probability density (HPD): 38.5 kya BP to 16.4 kya BP). Intriguingly, the analyses further revealed a deep split within the eastern (mixed) clade dating to about 23.0 kya BP (95% HPD: 33.3 kya BP to 15.7 kya BP), pointing to the possible existence of a not previously identified third evolutionary clade, comprising both some of our oldest samples, as well as a Thule period sample from Northwest Greenland and the Canadian Arctic Archipelago. We henceforth refer to this third clade as the northwestern clade or NW1. Our estimates of initial divergence within Atlantic walrus are in the younger range of that reported in previous studies and has a narrower confidence interval [31–34]. We suggest that our estimates are a better approximation of the true divergence times, due to our larger sample size representing most of the Atlantic walrus' range, the use of near-complete mitogenomes and the inclusion of ancient samples allowing for tip-dating in the phylogenetic analyses.

It has been hypothesized that Atlantic walrus survived the LGM in either one [35] or two refugia [33]. Our finding of three major clades separated by phylogenetic splits dating to the LGM could indicate that Atlantic walrus became isolated in not one or two, but three distinct glacial refugia from which they recolonized the Arctic as it deglaciated during the late Pleistocene and early Holocene. The location of such refugia will remain speculative. However, walrus palaeontological and/or zooarchaeological material dating to the period 50–20 kya BP has been recovered from several sites in the North Sea region [71–73], which suggest the presence of a population of walrus in the coastal areas of north-temperate Europe during the LGM. The existence of an Arctic marine ecosystem in the North Sea region during the late Pleistocene is supported by subfossil finds of multiple Arctic marine mammals, including ringed seals, polar bears and bowhead whales [12,73]. The oldest walrus finds in the Northwest Atlantic date to 12.0–9.7 kya BP, with a few zooarchaeological specimens from North Carolina and New Jersey in the USA, and the vast majority from the Canadian Maritimes region and the prehistoric Champlain Sea and the Canadian High Arctic [74–77]. By contrast, the oldest walrus finds from Greenland are younger at about 7.5 kya BP [19]. While not dating to the LGM, the large number of early Holocene finds and their geographical spread across the USA and Canadian coastlines does not contradict the hypothesized presence of glacial refugia in the northwest Atlantic; such finds could have been localized at the southern perimeter of the ice sheet or in sea-ice polynyas, pairing contemporary observations of wintering areas for contemporary walruses (e.g. the North Water polynya, [78]). In summary, we find support for the hypothesis that the Atlantic walrus survived the LGM in at least two distinct glacial refugia, which we, for now, propose were localized in the North Sea region and the Canadian Maritimes region. The origin of the third and previously undescribed Northwestern clade (NW1) is more complex. It could reflect an LGM refugia in Northwest Greenland-Arctic Archipelago, such as the North Water polynya, or it may correspond to an early migration at the time of the LGM by walrus into the region from the Northeast Atlantic, substantially preceding a second migratory wave from the northeast to the northwest about 5–4 kya (see below).

### (b) Holocene deglaciation drove further diversification of Atlantic walrus populations

**Figure 2.**
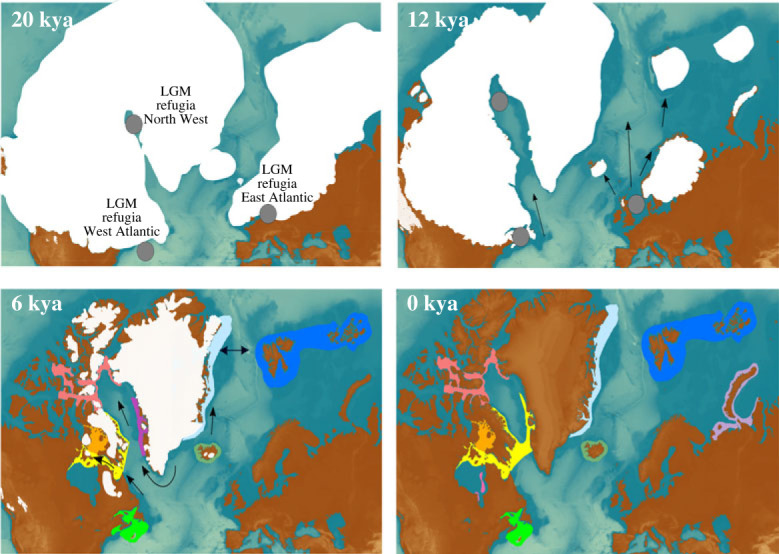
Hypothesized glacial refugia and Holocene expansion of walrus. Hypothesized LGM refugia for Atlantic walrus in the eastern, western and northwestern Atlantic (grey circles) and subsequent Holocene expansion and diversification (full arrows), coloured according to sample origins in the phylogeny. A 0 kya map reflects their current distribution based on [57,67,68,79,80]. The alternative to a Northwest refugia is an early migration from east to west (stippled arrow). Ice sheet time series adapted from [64–66] with basemaps from ETOPO1 Arc-Minute Global Relief Model available at https://www.ngdc.noaa.gov/mgg/global/ and modified using Inkscape (https://inkscape.org/). Additional time periods provided in electronic supplementary material, figure S3.

Our relatively large sample size of 82 ancient and historical walrus mitogenomes covering a period of nearly 8000 years allowed us to infer the biogeography of Atlantic walrus at finer spatio-temporal scales than previously possible (e.g. [33]). Indeed, our results provide a detailed chronology of the emergence and diversification of local Atlantic walrus populations that very precisely mirrors the likely contracting and isolating effects of LGM, as well as subsequent expansions during late Pleistocene and early Holocene deglaciation in the North Atlantic (figure 2; electronic supplementary material, figure S3). Specifically, the phylogenetic analyses suggest that the initial split into three clades—western, northwestern and eastern (mixed)—during the LGM was followed by diversification and expansion of the eastern (mixed) clade at roughly 15–12 kya BP during the warm Bølling-Allerød period and the onset of the Holocene that saw the gradual retreat of the Laurentide and Greenland Ice Sheets and associated sea-ice [10,81–83].

The now-extinct Icelandic walrus population in subclade E6 arose nearly 9 kya BP, coinciding with the deglaciation of Iceland [84], and fitting well with the earliest walrus finds in Iceland about 8.8 kya BP [34]. Curiously, two of the oldest Icelandic samples (XOR097 and XOR113) appear to have diverged much earlier at roughly 11 kya BP. This may indicate an early wave of colonizers and/or stray animals in Iceland, although so far not supported by zooarchaeological finds. The subclades E1, E2 and E5, comprising primarily East Greenland and Svalbard individuals, appeared between 8 and 5 kya BP as the warm Gulf Current had made these regions ice free [85,86].

In the western clade, our phylogenetic analyses revealed novel and remarkably fine-scale phylogeographic structure and chronology, pointing to: (i) the early diversification of walrus in the Canadian Maritimes (subclade W4) from other walruses in the western clade about 11 kya BP, (ii) the establishment of walrus populations in Foxe Basin and West Greenland (subclades W2–W3, as well as E3 and E7 in the eastern (mixed) clade) at roughly 6 kya BP, and (iii) the emergence of the Northwest Greenland subclade W1 at roughly 4 kya BP. Each of these splits into distinct populations are remarkably well-supported (100%) in the phylogeny, providing stronger support for their genetic uniqueness than presented in previous studies [33,80,87]. Together, this indicates that walrus likely moved north from a southern refugium e.g. in the Canadian Maritimes to colonize and diversify in the greater Davis Strait region as this deglaciated around 12 kya BP [88,89]. Subsequently, walruses tracked available habitat north and west into the Foxe Channel, Foxe Basin and Northwest Greenland-Canadian Arctic Archipelago when these regions deglaciated between 8.5 and 6 kya BP [90–94].

**Figure 3.**
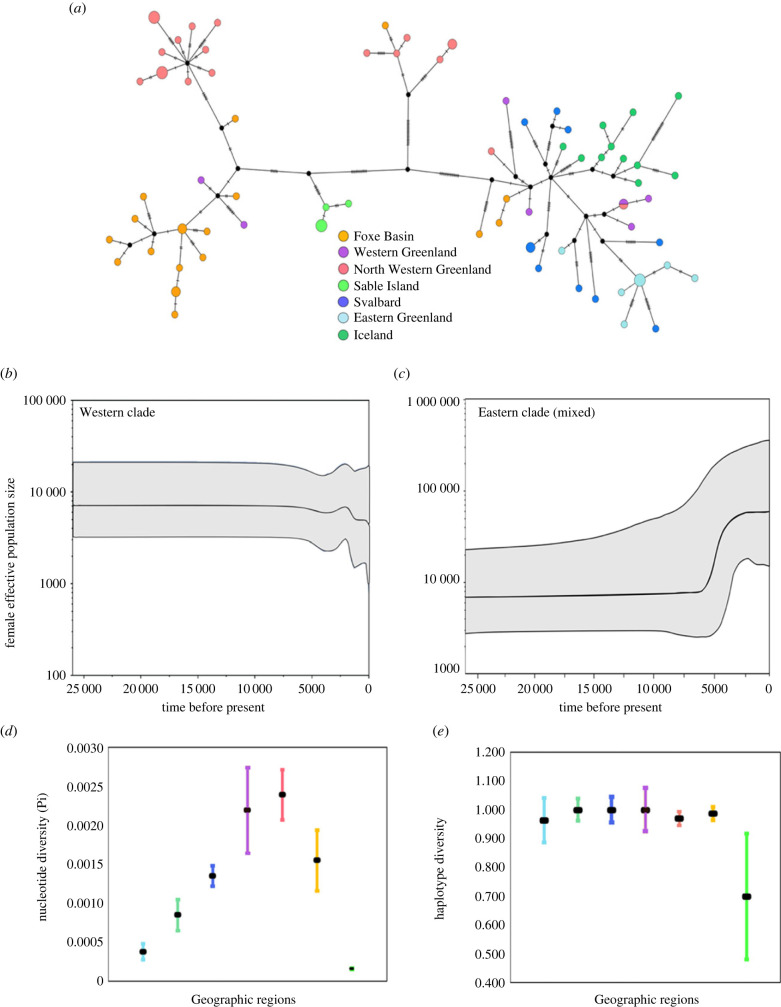
Demographic history and genetic diversity in the Atlantic walrus. (*a*) Haplotype network of 82 ancient and historical walrus mitogenomes. Circles are sized according to the number of individuals sharing the same haplotype, and the number of mutations between each haplotype is defined by the hatched lines. (*b,c*) Bayesian skyline plots (BSP) for the western clade (*n* = 36) and for the eastern clade (*n* = 39), respectively. (*d*,*e*) Mitochondrial nucleotide and haplotype diversity estimated for each of the seven main walrus populations. Error bars indicate standard deviations. Colours in *a*, *d* and *e* reflect geographical origin.

The division into multiple distinct ancient Atlantic walrus populations is supported by high and statistically significantly *F*_ST_ and *d*_A_ values for almost all pairwise population comparisons (electronic supplementary material, table S5). A high level of genetic structure among ancient walrus populations was also observed in the haplotype network (figure 3*a*), and corresponds well with genetic analyses of contemporary walruses [30,31,35,57], although our mitogenomes revealed a more detailed level of phylogeographic structure.

The existence of such multiple genetically distinct and geographically localized maternal (i.e. mitogenome) lineages is unique among the Arctic marine mammals studied to date, which typically comprise two to three genetic populations or evolutionary clades per species, with no clear geographical pattern in the North Atlantic (e.g. [15,16,20]). The long-term persistence of multiple walrus populations after their initial establishment during the early Holocene may be the result of a high level of maternal site-fidelity to feeding grounds on shallow-water mollusc banks, as well as breeding grounds in localized polynyas [26], minimizing the genetic admixture of different populations. Still, we note the presence of western animals within the eastern (mixed) genetic clade (e.g. subclades E3 and E7), which could result from western populations occasionally receiving eastern migrants following prevailing East Greenland and West Greenland currents and associated ice-flow [83], as also suggested in [33]. That is, the occurrence of western animals in both the western and eastern (mixed) clade could reflect multiple colonization events into the western regions (i.e. West Greenland, Foxe Basin and Northwest Greenland); first one or two colonization events from the Northeast Atlantic giving rise to subclades E3 and E7 and perhaps NW1, and later a massive colonization wave from the hypothesized refugia in the Canadian Maritimes giving rise to subclades W1–W3 in the western clade.

### (c) Later impacts of environmental change and human exploitation

In addition to the inferred Late Pleistocene and Early Holocene diversification, the effective population size of Atlantic walrus may have been affected by more recent climate change (e.g. Roman Warm Period (RWP) and Little Ice Age (LIA)) and overexploitation [33,34,80,95]. To explore the demographic history of Atlantic walrus, we constructed BSP (figure 3*b,c*) for the western and eastern Atlantic walrus clades, respectively, and estimated levels of haplotype and nucleotide diversity for each of the inferred populations within each clade (figure 3*d,e*; electronic supplementary material, table S6). The BSPs revealed very little change in post-LGM effective population size in the western clade until a slight population size decrease at 2–1 kya BP (figure 3*b,c*). Conversely, the BSP showed a signature of population expansion in the eastern clade, starting 10 kya and levelling off about 3 kya; an expansion also detected by Andersen *et al.* [31] using genetic data from contemporary walrus populations. This signature of expansion may reflect the recolonization of the northeast Atlantic from the hypothesized North Sea LGM-refugia. Overall, levels of haplotype diversity were similar across populations (with the exception of Sable Island), whereas levels of nucleotide diversity were higher for the populations in West Greenland and Arctic Canada (figure 3*d,e*; electronic supplementary material, table S3); these higher levels of diversity could be driven by an influx of eastern clade haplotypes into western populations, as discussed above. In contemporary populations in East Greenland and Svalbard, the levels of genetic diversity reported for mtDNA d-loop data [27,31] are comparable to those we estimated, supporting the BSP analysis in suggesting no major declines in genetic diversity in the eastern clade.

The lack of detectable declines in effective population size and genetic diversity in the eastern clade is surprising given the substantial Norse exploitation of walruses and complete eradication of populations in Iceland [33,34,96]. The estimated decline at 2–1 kya in the western clade overlaps partly with the early period of Norse walrus exploitation [33,95], but predates exploitation and eradication of walrus in the Canadian Maritimes by the Basque and other European whalers [80]. The western decline also coincides with the RWP [97,98], which had profound impacts on the North Atlantic marine ecosystems, including the distribution and abundance of bivalve molluscs *Hiatella* sp. and *Macoma* sp. [99,100]. These are among the walrus' preferred prey species [101–103], and any impacts on them during RWP might in turn have indirectly affected the abundance and distribution of walrus. Alternatively, simulation studies suggest that the existence of population structure may lead to false signals of population decline [104], which could account for the population decline detected in the western clade. However, if so, we would have expected an even larger bias in the eastern clade, which arguably is more admixed. Determining the full effect of historic and recent exploitation and climatic warm periods on walrus genetic diversity may require larger sample sizes and the addition of nuclear genome data from both ancient, historic and contemporary populations.

### (d) Conclusion and perspectives

Our results show that large-scale climate fluctuations related to the LGM and subsequent deglaciation of the Arctic were strong and rapid drivers of Atlantic walrus mitogenome diversification. The data provide evidence for a highly geographical pattern of Atlantic walrus diversification and likely multiple waves of expansion into the Arctic followed by isolation, contradicting the patterns so far reported for Arctic pinnipeds and many other Arctic marine species. We find support for a *tabula rasa* scenario with two low-latitude glacial refugia on each side of the North Atlantic establishing Arctic walrus populations. Further, our results hint at the existence of a previously undescribed evolutionary clade, possibly reflecting a high-latitude refugia in northwestern Greenland or Canadian Arctic Archipelago, corresponding to a marine equivalent of the terrestrial *nunatak* scenario. Overall, together with other studies of Arctic marine mammals our study highlights that responses to climate change are highly species specific and geographically dependent, even within a relatively narrow ecological and evolutionary assemblage of species. Moreover, as we show, the micro-evolutionary response to environmental change can occur very rapidly and may leave profound marks on genetic diversity and differentiation; an observation with potential direct parallels to the effects of ongoing and future Arctic warming on the diversity and connectivity of Arctic marine species and populations.

Further studies of Atlantic walrus, and other Arctic marine species, should expand the spatio-temporal coverage to include samples from all parts of the Arctic, as well as from ancient specimens retrieved from past high- and low-latitude glacial refugia. The addition of nuclear genome data will likely increase the resolution of demographic simulations, and reveal patterns not detected by the maternally inherited mitogenome, such as male-mediated gene-flow and local adaptation. Furthermore, detailed insights on the role of past ecosystem dynamics, including putative shifts in trophic networks, may be obtained by generating stable isotope data, allowing for a more holistic understanding of the likely multifaceted effects of environmental change on Arctic marine ecosystems. Such data will be of relevance for management and conservation efforts attempting to anticipate and mitigate the impacts of a future ice-free Arctic on marine species, ecosystems and associated livelihoods, including Arctic Indigenous communities.

## Data Availability

Holocene deglaciation drove rapid genetic diversification of Atlantic walrus input files: Dryad https://doi.org/10.5061/dryad.qbzkh18pp [105]. The data are provided in electronic supplementary material [106].

## References

[1] Cooley S *et al*. 2022 Oceans and coastal ecosystems and their services. In *Climate Change 2022: Impacts, Adaptation and Vulnerability*. Contribution of Working Group II to the Sixth Assessment Report of the Intergovernmental Panel on Climate Change (eds H-O Pörtner, DC Roberts, M Tignor, ES Poloczanska, K Mintenbeck, A Alegría, M Craig, S Langsdorf, S Löschke, V Möller, A Okem, B Rama) pp. 379-550. Cambridge, UK and New York, NY: Cambridge University Press. (https://www.researchgate.net/publication/359668711_Oceans_and_Coastal_Ecosystems_and_their_Services)

[2] Evans PGH, Pierce GP, Panigada S. 2010 Climate change and marine mammals. J. Mar. Biol. Assoc. UK **90**, 1483-1487. (10.1017/S0025315410001815)

[3] Wassmann P, Duarte CM, Agustí S, Sejr MK. 2011 Footprints of climate change in the Arctic marine ecosystem. Glob. Change Biol. **17**, 1235-1249. (10.1111/j.1365-2486.2010.02311.x)

[4] Doney SC et al. 2012 Climate change impacts on marine ecosystems. Annu. Rev. Mar. Sci. **4**, 11-37. (10.1146/annurev-marine-041911-111611) 22457967

[5] Frainer A, Primicerio R, Kortsch S, Aune M, Dolgov AV, Fossheim M, Aschan MM. 2017 Climate-driven changes in functional biogeography of Arctic marine fish communities. Proc. Natl Acad. Sci. USA **114**, 12 202-12 207. (10.1073/pnas.1706080114) PMC5699037 29087943

[6] Laidre KL, Heide-Jørgensen MP. 2012 Spring partitioning of Disko Bay, West Greenland, by Arctic and Subarctic baleen whales. ICES J. Mar. Sci. **69**, 1226-1233. (10.1093/icesjms/fss095)

[7] Hamilton CD, Lydersen C, Ims RA, Kovacs KM. 2015 Predictions replaced by facts: a keystone species' behavioural responses to declining Arctic sea-ice. Biol. Lett. **11**, 20150803. (10.1098/rsbl.2015.0803) 26582841 PMC4685547

[8] VanWormer E et al. 2019 Viral emergence in marine mammals in the North Pacific may be linked to Arctic sea ice reduction. Sci. Rep. **9**, 15569. (10.1038/s41598-019-51699-4) 31700005 PMC6838065

[9] Kelly BP, Whiteley A, Tallmon D. 2010 The Arctic melting pot. Nature **468**, 891. (10.1038/468891a) 21164461

[10] Clark PU, Dyke AS, Shakun JD, Carlson AE, Clark J, Wohlfarth B, Mitrovica JX, Hostetler SW, McCabe AM. 2009 The last glacial maximum. Science **325**, 710-714. (10.1126/science.1172873) 19661421

[11] Laidre KL et al. 2015 Arctic marine mammal population status, sea ice habitat loss, and conservation recommendations for the 21st century: Arctic Marine Mammal Conservation. Conserv. Biol. **29**, 724-737. (10.1111/cobi.12474) 25783745 PMC5008214

[12] Foote AD et al. 2013 Ancient DNA reveals that bowhead whale lineages survived Late Pleistocene climate change and habitat shifts. Nat. Commun. **4**, 1677. (10.1038/ncomms2714) 23575681

[13] Palsbøll PJ, Heide-Jørgensen MP, Dietz R. 1997 Population structure and seasonal movements of narwhals, *Monodon monoceros*, determined from mtDNA analysis. Heredity **78**, 284-292. (10.1038/hdy.1997.43) 9119704

[14] Westbury MV, Petersen B, Garde E, Heide-Jørgensen MP, Lorenzen ED. 2019 Narwhal genome reveals long-term low genetic diversity despite current large abundance size. iScience **15**, 592-599. (10.1016/j.isci.2019.03.023) 31054839 PMC6546971

[15] Louis M et al. 2020 Influence of past climate change on phylogeography and demographic history of narwhals, *Monodon monoceros*. Proc. R. Soc. B **287**, 20192964. (10.1098/rspb.2019.2964) PMC7211449 32315590

[16] Skovrind M, Castruita JAS, Madsen TB, Postma L, Lorenzen ED. 2019 Patterns of mtDNA variation in relation to currently recognized stocks of beluga whales, Delphinapterus leucas. Mar. Fish. Rev. **81**, 87-98.

[17] Liu S et al. 2014 Population genomics reveal recent speciation and rapid evolutionary adaptation in polar bears. Cell **157**, 785-794. (10.1016/j.cell.2014.03.054) 24813606 PMC4089990

[18] Lan T et al. 2022 Insights into bear evolution from a Pleistocene polar bear genome. In bioRxiv (p. 2021.12.11.472228). (10.1101/2021.12.11.472228)

[19] Bennike O, Wagner B. 2021 Quaternary vertebrates from the North Atlantic Islands. In Biogeography in the sub-Arctic, pp. 147-160. Hoboken, NJ: Wiley.

[20] Carr SM, Duggan AT, Stenson GB, Marshall HD. 2015 Quantitative phylogenomics of within-species mitogenome variation: Monte Carlo and non-parametric analysis of phylogeographic structure among discrete transatlantic breeding areas of harp seals (*Pagophilus groenlandicus*). PLoS ONE **10**, e0134207. (10.1371/journal.pone.0134207) 26301872 PMC4547794

[21] Olsen MT, Volny VH, Bérubé M, Dietz R, Lydersen C, Kovacs KM, Dodd RS, Palsbøll PJ. 2011 A simple route to single-nucleotide polymorphisms in a nonmodel species: identification and characterization of SNPs in the Arctic ringed seal (*Pusa hispida hispida*). Mole. Ecol. Resour. **11**(Suppl. 1), 9-19. (10.1111/j.1755-0998.2010.02941.x) 21429159

[22] Maggs CA et al. 2008 Evaluating signatures of glacial refugia for North Atlantic benthic marine taxa. Ecology **89**(11 Suppl), S108-S122. (10.1890/08-0257.1) 19097488

[23] Hardy SM, Carr CM, Hardman M, Steinke D, Corstorphine E, Mah C. 2011 Biodiversity and phylogeography of Arctic marine fauna: insights from molecular tools. Mar. Biodivers. **41**, 195-210.

[24] Bringloe TT, Verbruggen H, Saunders GW. 2020 Unique biodiversity in Arctic marine forests is shaped by diverse recolonization pathways and far northern glacial refugia. Proc. Natl Acad. Sci. USA **117**, 22 590-22 596. (10.1073/pnas.2002753117) PMC7486700 32843343

[25] Jacobsen MW et al. 2021 A melting pot in the Arctic: analysis of mitogenome variation in Arctic char (*Salvelinus alpinus*) reveals a 1000-km contact zone between highly divergent lineages. Ecol. Freshwater Fish **31**, 330-346. (10.1111/eff.12633)

[26] Born EW, Wiig Ø. 2021 Ecology and behavior of Atlantic walruses. In The Atlantic Walrus, (eds X Keighley, MT Olsen, P Jordan, S Desjardins), pp. 39-76. Academic Press. (10.1016/b978-0-12-817430-2.00001-7)

[27] Lindqvist C, Bachmann L, Andersen LW, Born EW, Arnason U, Kovacs KM, Lydersen C, Abramov AV, Wiig Ø. 2009 The Laptev Sea walrus *Odobenus rosmarus laptevi*: an enigma revisited. Zool. Scrip. **38**, 113-127. (10.1111/j.1463-6409.2008.00364.x)

[28] Sonsthagen SA, Jay CV, Fischbach AS, Sage GK, Talbot SL. 2012 Spatial genetic structure and asymmetrical gene flow within the Pacific walrus. J. Mammalogy **93**, 1512-1524. (10.1644/11-MAMM-A-344.1)

[29] Beatty WS, Lemons PR, Sethi SA, Everett JP, Lewis CJ, Lynn RJ, Cook GM, Garlich-Miller JL, Wenburg JK. 2020 Panmixia in a sea ice-associated marine mammal: evaluating genetic structure of the Pacific walrus (*Odobenus rosmarus divergens*) at multiple spatial scales. J. Mammalogy **101**, 755-765. (10.1093/jmammal/gyaa050)

[30] Andersen LW, Born EW, Gjertz I, Wiig O, Holm LE, Bendixen C. 1998 Population structure and gene flow of the Atlantic walrus (*Odobenus rosmarus rosmarus*) in the eastern Atlantic Arctic based on mitochondrial DNA and microsatellite variation. Mol. Ecol. **7**, 1323-1336. (10.1046/j.1365-294x.1998.00455.x) 9787444

[31] Andersen LW, Jacobsen MW. 2017 Walruses (*Odobenus rosmarus rosmarus*) in the Pechora Sea in the context of contemporary population structure of Northeast Atlantic walruses. Biol. J. Linn. Soc. **122**, 897-915. (10.1093/biolinnean/blx093)

[32] Shafer ABA, Gattepaille LM, Stewart REA, Wolf JBW. 2014 Demographic inferences using short-read genomic data in an approximate Bayesian computation framework: in silico evaluation of power, biases and proof of concept in Atlantic walrus. Mol. Ecol. **24**, 328-345. (10.1111/mec.13034) 25482153

[33] Star B, Barrett JH, Gondek AT, Boessenkool S. 2018 Ancient DNA reveals the chronology of walrus ivory trade from Norse Greenland. Proc. R. Soc. B **285**, 20180978. (10.1098/rspb.2018.0978) PMC6111184 30089624

[34] Keighley X, Pálsson S, Einarsson BF, Petersen A, Fernández-Coll M, Jordan P, Olsen MT, Malmquist HJ. 2019 Disappearance of Icelandic walruses coincided with Norse settlement. Mol. Biol. Evol. **36**, 2656-2667. (10.1093/molbev/msz196) 31513267 PMC6878957

[35] Born E, Andersen L, Gjertz I, Wiig Ø. 2001 A review of the genetic relationships of Atlantic walrus (*Odobenus rosmarus rosmarus*) east and west of Greenland. Polar Biol. **24**, 713-718. (10.1007/s003000100277)

[36] Cooper A, Poinar HN. 2000 Ancient DNA: do it right or not at all. Science **289**, 1139. (10.1126/science.289.5482.1139b) 10970224

[37] Gilbert MTP, Bandelt H-J, Hofreiter M, Barnes I. 2005 Assessing ancient DNA studies. Trends Ecol. Evol. **20**, 541-544. (10.1016/j.tree.2005.07.005) 16701432

[38] Dabney J et al. 2013 Complete mitochondrial genome sequence of a Middle Pleistocene cave bear reconstructed from ultrashort DNA fragments. Proc. Natl Acad. Sci. USA **110**, 15 758-15 763. (10.1073/pnas.1314445110) PMC3785785 24019490

[39] Boessenkool S, Hanghøj K, Nistelberger HM, Der Sarkissian C, Gondek AT, Orlando L, Barrett JH, Star B. 2017 Combining bleach and mild predigestion improves ancient DNA recovery from bones. Mole. Ecol. Resour. **17**, 742-751. (10.1111/1755-0998.12623) 27790833

[40] Carøe C, Gopalakrishnan S, Vinner L, Mak SST, Sinding MHS, Samaniego JA, Wales N, Sicheritz-Pontén T, Gilbert MTP. 2018 Single-tube library preparation for degraded DNA. British Ecol. Soc. **9**, 410-419. (10.1111/2041-210X.12871)

[41] Barnett R et al. 2018 No longer locally extinct? Tracing the origins of a lion (*Panthera leo*) living in Gabon. Conserv. Genetics **19**, 611-618. (10.1007/s10592-017-1039-2) PMC6448349 31007636

[42] van der Valk T, Vezzi F, Ormestad M, Dalén L, Guschanski K. 2020 Index hopping on the Illumina HiseqX platform and its consequences for ancient DNA studies. Molecular Ecology Resources **20**, 1171-1181. (10.1111/1755-0998.13009) 30848092

[43] Schubert M et al. 2014 Characterization of ancient and modern genomes by SNP detection and phylogenomic and metagenomic analysis using PALEOMIX. Nat. Protoc. **9**, 1056-1082. (10.1038/nprot.2014.063) 24722405

[44] Arnason U et al. 2002 Mammalian mitogenomic relationships and the root of the eutherian tree. Proc. Natl Acad. Sci. USA **99**, 8151-8156. (10.1073/pnas.102164299) 12034869 PMC123036

[45] Li H, Handsaker B, Wysoker A, Fennell T, Ruan J, Homer N, Marth G, Abecasis G, Durbin R and 1000 Genome Project Data Processing Subgroup. 2009 The sequence alignment/map format and SAMtools. Bioinformatics **25**, 2078-2079. (10.1093/bioinformatics/btp352) 19505943 PMC2723002

[46] Li H, Durbin R. 2009 Fast and accurate short read alignment with Burrows–Wheeler transform. Bioinformatics **25**, 1754-1760. (10.1093/bioinformatics/btp324) 19451168 PMC2705234

[47] Ginolhac A, Rasmussen M, Gilbert MTP, Willerslev E, Orlando L. 2011 mapDamage: testing for damage patterns in ancient DNA sequences. Bioinformatics **27**, 2153-2155. (10.1093/bioinformatics/btr347) 21659319

[48] Schubert M, Lindgreen S, Orlando L. 2016 AdapterRemoval v2: rapid adapter trimming, identification, and read merging. BMC Res. Notes **9**, 88. (10.1186/s13104-016-1900-2) 26868221 PMC4751634

[49] Korneliussen TS, Albrechtsen A, Nielsen R. 2014 ANGSD: analysis of next generation sequencing data. BMC Bioinf. **15**, 356. (10.1186/s12859-014-0356-4) PMC4248462 25420514

[50] Li H. 2011 Improving SNP discovery by base alignment quality. Bioinformatics **27**, 1157-1158. (10.1093/bioinformatics/btr076) 21320865 PMC3072548

[51] Rozas J, Ferrer-Mata A, Sánchez-DelBarrio JC, Guirao-Rico S, Librado P, Ramos-Onsins SE, Sánchez-Gracia A. 2017 DnaSP 6: DNA sequence polymorphism analysis of large data sets. Mol. Biol. Evol. **34**, 3299-3302. (10.1093/molbev/msx248) 29029172

[52] Excoffier L, Lischer HE. 2010 Arlequin suite ver 3.5: a new series of programs to perform population genetics analyses under Linux and Windows. Mole. Ecol. Resour. **10**, 54-567. (10.1111/j.1755-0998.2010.02847.x) 21565059

[53] Leigh JW, Bryant D. 2015 Popart: full-feature software for haplotype network construction. Methods Ecol. Evol. **6**, 1110-1116. (10.1111/2041-210X.12410)

[54] Trifinopoulos J, Nguyen L-T, von Haeseler A, Minh BQ. 2016 W-IQ-TREE: a fast online phylogenetic tool for maximum likelihood analysis. Nucleic Acids Res. **44**(W1), W232-W235. (10.1093/nar/gkw256) 27084950 PMC4987875

[55] Rambaut A, Lam TT, Carvalho ML, Pybus OG. 2016 Exploring the temporal structure of heterochronous sequences using TempEst (formerly Path-O-Gen). Virus Evol. **2**, vew007. (10.1093/ve/vew007) 27774300 PMC4989882

[56] Lanfear R, Frandsen PB, Wright AM, Senfeld T, Calcott B. 2017 PartitionFinder 2: new methods for selecting partitioned models of evolution for molecular and morphological phylogenetic analyses. Mol. Biol. Evol. **34**, 772-773. 28013191 10.1093/molbev/msw260

[57] Andersen LW, Born EW, Doidge DW, Gjertz I, Wiig Ø, Waples RS. 2009 Genetic signals of historic and recent migration between sub-populations of Atlantic walrus *Odobenus rosmarus rosmarus* west and east of Greenland. Endangered Species Res. **9**, 197-211. (10.3354/esr00242)

[58] Drummond AJ, Ho SYW, Phillips MJ, Rambaut A. 2006 Relaxed phylogenetics and dating with confidence. PLoS Biol. **4**, e88. (10.1371/journal.pbio.0040088) 16683862 PMC1395354

[59] Pybus OG. 2006 Model selection and the molecular clock. PLoS Biol. **4**, e151. (10.1371/journal.pbio.0040151) 16683863 PMC1459243

[60] Bouckaert RR et al. 2019 BEAST 2.5: an advanced software platform for Bayesian evolutionary analysis. PLoS Comput. Biol. **15**, e1006650. (10.1371/journal.pcbi.1006650) 30958812 PMC6472827

[61] Rambaut A, Drummond AJ. 2018 TreeAnnotator v2.5.1. Available as part of the BEAST package at http://beast.bio.ed.ac.uk.

[62] Rambaut A. 2012 FigTree v1. 4. See http://tree.bio.ed.ac.uk/software/figtree/.

[63] Environmental Systems Research Institute (ESRI). 2012 ArcGIS Release 10.1. Redlands, CA.

[64] Dyke AS, Moore AJ, Robertson L. 2003 Deglaciation of North America. Ottawa, Ontario, Canada: Geological Survey of Canada.

[65] Hughes ALC, Gyllencreutz R, Lohne ØS, Mangerud J, Svendsen JI. 2016 The last Eurasian ice sheets – a chronological database and time-slice reconstruction, DATED-1. Boreas **45**, 1-45. (10.1111/bor.12142)

[66] Batchelor CL, Margold M, Krapp M, Murton DK, Dalton AS, Gibbard PL, Stokes CR, Murton JB, Manica A. 2019 The configuration of Northern Hemisphere ice sheets through the quaternary. Nat. Commun. **10**, 3713. (10.1038/s41467-019-11601-2) 31420542 PMC6697730

[67] Andersen LW, Born EW, Stewart REA, Dietz R, Doidge DW, Lanthier C. 2014 A genetic comparison of West Greenland and Baffin Island (Canada) walruses: management implications **9**, 33-52.

[68] Born EW, Gjertz I, Reeves RR. 1995 Population assessment of Atlantic walrus (*Odobenus rosmarus rosmarus* L.). Population Assessment of Atlantic Walrus. Norsk Polarinstitutts Meddelelser **138**, 100.

[69] Inkscape Project. 2020. Inkscape. Retrieved from https://inkscape.org.

[70] Rieux A, Balloux F. 2016 Inferences from tip-calibrated phylogenies: a review and a practical guide. Mol. Ecol. **25**, 1911-1924. (10.1111/mec.13586) 26880113 PMC4949988

[71] Møhl U. 1985 The walrus, *Odobenus rosmarus* (L.), as a ‘Danish’ faunal element during the Weichsel Ice Age. Bull. Geol. Soc. Den. **34**, 83-85. (10.37570/bgsd-1985-34-08)

[72] Post K. 2005 *A Weichselian marine mammal assemblage from the southern North Sea*. DEINSEA 11:21–27.

[73] Aaris-Sørensen K. 2009 Diversity and dynamics of the mammalian fauna in Denmark throughout the last glacial-interglacial cycle, 115-0 kyr BP. Fossils and strata 57, pp. 1-59. Oslo; Copenhagen; Stockholm: Scandinavian University Press.

[74] Miller RF. 1990 New records of postglacial walrus and a review of quaternary marine mammals in New Brunswick. Atlantic Geosci. **26**, 1. (10.4138/1695)

[75] Miller R. 1997 New records and AMS radiocarbon dates on Quaternary Walrus (*Odobenus Rosmarus*) from New Brunswick. Géographie Phys. Quaternaire **51**, 107-111. (10.7202/004852ar)

[76] Dyke AS, Hooper J, Harington CR, Savelle JM. 1999 The Late Wisconsinan and Holocene Record of Walrus (*Odobenus rosmarus*) from North America: a review with new data from Arctic and Atlantic Canada. Arctic **52**, 160-181.

[77] Feranec RS, Cournoyer ME, Kozlowski AL. 2021 14C dates and stable isotope ecology of marine vertebrates in the late pleistocene-early Holocene Champlain Sea. Radiocarbon **63**, 1259-1272. (10.1017/RDC.2021.40)

[78] Heide-Jørgensen MP, Sinding M-HS, Nielsen NH, Rosing-Asvid A, Hansen RG. 2016 Large numbers of marine mammals winter in the North Water polynya. Polar Biol. **39**, 1605-1614. (10.1007/s00300-015-1885-7)

[79] Dietz et al 2014. Movements of walruses (*Odobenus rosmarus*) between Central West Greenland and Southeast Baffin Island, 2005-2008. NAMMCO Scientific Publications **9**, 53-74. (10.7557/3.2605)

[80] McLeod BA, Frasier TR, Lucas Z. 2014 Assessment of the extirpated Maritimes walrus using morphological and ancient DNA analysis. PLoS ONE **9**, e99569. (10.1371/journal.pone.0099569) 24924490 PMC4055739

[81] Pflaumann U et al. 2003 Glacial North Atlantic: sea-surface conditions reconstructed by GLAMAP 2000. Paleoceanography **18**, 1-28. (10.1029/2002pa000774)

[82] Carlson AE, LeGrande AN, Oppo DW, Came RE, Schmidt GA, Anslow FS, Licciardi JM, Obbink EA. 2008 Rapid early Holocene deglaciation of the Laurentide ice sheet. Nat. Geosci. **1**, 620-624. (10.1038/ngeo285)

[83] Sheldon C, Jennings A, Andrews JT, Ó Cofaigh C, Hogan K, Dowdeswell JA, Seidenkrantz M-S. 2016 Ice stream retreat following the LGM and onset of the west Greenland current in Uummannaq Trough, west Greenland. Quat. Sci. Rev. **147**, 27-46. (10.1016/j.quascirev.2016.01.019)

[84] Ingólfsson Ó, Norðdahl H, Schomacker A. 2010. Deglaciation and Holocene glacial history of Iceland. Developments in quaternary sciences. **13**, 51-68.

[85] Landvik JY, Bondevik S, Elverhøi A, Fjeldskaar W, Mangerud J, Salvigsen O, Siegert MJ, Svendsen J-I, Vorren TO. 1998 The last glacial maximum of Svalbard and the Barents Sea area: ice sheet extent and configuration. Quat. Sci. Rev. **17**, 43-75. (10.1016/S0277-3791(97)00066-8)

[86] Van Vliet-Lanoë B, Guðmundsson Å, Guillou H, Duncan RA, Genty D, Ghaleb B, Gouy S, Récourt P, Scaillet S. 2007 Limited glaciation and very early deglaciation in central Iceland: implications for climate change. Comptes Rendus: Geosci. **339**, 1-12. (10.1016/j.crte.2006.12.001)

[87] Andersen LW, Born EW. 2000 Indications of two genetically different subpopulations of Atlantic walruses (*Odobenus rosmarus rosmarus*) in west and northwest Greenland. Canad. J. Zool. **78**, 1999-2009. (10.1139/z00-118)

[88] Briner JP et al. 2016 Holocene climate change in Arctic Canada and Greenland. Quat. Sci. Rev. **147**, 340-364. (10.1016/j.quascirev.2016.02.010)

[89] Hogan KA, Ó Cofaigh C, Jennings AE, Dowdeswell JA, Hiemstra JF. 2016 Deglaciation of a major palaeo-ice stream in Disko Trough, West Greenland. Quat. Sci. Rev. **147**, 5-26. (10.1016/j.quascirev.2016.01.018)

[90] Dyke AS. 2004 An outline of North American deglaciation with emphasis on central and northern Canada. Quaternary glaciations-extent and chronology - part II: North America, 373-424.

[91] Vickers KJ, Ward BC, Utting DJ, Telka AM. 2010 Deglacial reservoir age and implications, Foxe Peninsula, Baffin Island. J. Quat. Sci. **25**, 1338-1346.

[92] Ross M, Utting DJ, Lajeunesse P, Kosar KGA. 2012 Early Holocene deglaciation of northern Hudson Bay and Foxe Channel constrained by new radiocarbon ages and marine reservoir correction. Quat. Res. **78**, 82-94. (10.1016/j.yqres.2012.03.001)

[93] Georgiadis E, Giraudeau J, Martinez P, Lajeunesse P, St-Onge G, Schmidt S, Massé G. 2018 Deglacial to postglacial history of Nares Strait, Northwest Greenland: a marine perspective from Kane Basin. Clim. Past **14**, 1991-2010.

[94] Ceperley EG, Marcott SA, Reusche MM, Barth AM, Mix AC, Brook EJ, Caffee M. 2020 Widespread early Holocene deglaciation, Washington Land, northwest Greenland. Quat. Sci. Rev. **231**, 106181. (10.1016/j.quascirev.2020.106181)

[95] Barrett JH, Boessenkool S, Kneale CJ. 2020 Ecological globalisation, serial depletion and the medieval trade of walrus rostra. Quat. Sci. Rev. **229**, 106122. (10.1016/j.quascirev.2019.106122)

[96] Barrett JH, Khamaiko N, Ferrari G, Cuevas A, Kneale C, Hufthammer AK, Pálsdóttir AH, Star B. 2022 Walruses on the Dnieper: new evidence for the intercontinental trade of Greenlandic ivory in the Middle Ages. Proc. R. Soc. B **289**, 20212773. (10.1098/rspb.2021.2773) PMC8984804 35382600

[97] Patterson WP, Dietrich KA, Holmden C, Andrews JT. 2010 Two millennia of North Atlantic seasonality and implications for Norse colonies. Proc. Natl Acad. Sci. USA **107**, 5306-5310. (10.1073/pnas.0902522107) 20212157 PMC2851789

[98] Kjær KH et al. 2022 Glacier response to the Little Ice Age during the Neoglacial cooling in Greenland. Earth Sci. Rev. **227**, 103984. (10.1016/j.earscirev.2022.103984)

[99] Laakkonen HM, Strelkov P, Väinölä R. 2015 Molecular lineage diversity and inter-oceanic biogeographical history in *Hiatella* (Mollusca, Bivalvia). Zool. Scr. **44**, 383-402. (10.1111/zsc.12105)

[100] Arivalagan J, Marie B, Sleight VA, Clark MS, Berland S, Marie A. 2016 Shell matrix proteins of the clam, Mya truncata: roles beyond shell formation through proteomic study. Mar. Genomics **27**, 69-74. (10.1016/j.margen.2016.03.005) 27068305

[101] Gjertz I, Wiig Ø. 1992 Feeding of walrus *Odobenus rosmarus* in Svalbard. Polar Rec. **28**, 57-59. (10.1017/S0032247400020283)

[102] Born EW, Rysgaard S, Ehlmé G, Sejr M, Acquarone M, Levermann N. 2003 Underwater observations of foraging free-living Atlantic walruses (*Odobenus rosmarus rosmarus*) and estimates of their food consumption. Polar Biol. **26**, 348-357. (10.1007/s00300-003-0486-z)

[103] Denisenko SG, Denisenko NV, Chaban EM, Gagaev SY, Petryashov VV, Zhuravleva NE, Sukhotin AA. 2019 The current status of the macrozoobenthos around the Atlantic walrus haul-outs in the Pechora Sea (SE Barents Sea). Polar Biol. **42**, 1703-1717. (10.1007/s00300-018-02455-3)

[104] Heller R, Chikhi L, Siegismund HR. 2013 The confounding effect of population structure on Bayesian skyline plot inferences of demographic history. PLoS ONE **8**, e62992. (10.1371/journal.pone.0062992) 23667558 PMC3646956

[105] Ruiz-Puerta E et al. 2023 Data from: Holocene deglaciation drove rapid genetic diversification of Atlantic walrus. Dryad Digital Repository. (10.5061/dryad.qbzkh18pp) PMC10523089 37752842

[106] Ruiz-Puerta E et al. 2023 Holocene deglaciation drove rapid genetic diversification of Atlantic walrus. Figshare. (10.6084/m9.figshare.c.6825679) PMC10523089 37752842

